# Clinical and Molecular Spectrum of *DDX41* Variants in Korean Patients with Hematologic Malignancies

**DOI:** 10.3390/jcm14227999

**Published:** 2025-11-11

**Authors:** Boram Kim, Dae-Ho Choi, Jun Ho Jang, Chul Won Jung, Hee-Jin Kim, Hyun-Young Kim

**Affiliations:** 1 Department of Laboratory Medicine and Genetics, Samsung Medical Center, Sungkyunkwan University School of Medicine, Seoul 06351, Republic of Korea; kbrinlab@gmail.com (B.K.);; 2 Department of Laboratory Medicine, Korea University Guro Hospital, Korea University College of Medicine, Seoul 08308, Republic of Korea; 3 Division of Hematology-Oncology, Department of Medicine, Samsung Medical Center, Sungkyunkwan University School of Medicine, Seoul 06351, Republic of Korea

**Keywords:** *DDX41*, germline variant, somatic variant, germline predisposition, hematologic malignancy

## Abstract

**Background:** *DDX41* is one of the most frequent adult-onset myeloid neoplasm predisposition gene, yet its biology remains incompletely defined. This study investigated the frequency, spectrum, and clinical characteristics of *DDX41* variants in Korean patients with hematologic malignancies, including the patterns of co-occurring somatic variants. **Methods:** We retrospectively reviewed 716 patients with hematologic malignancies who underwent targeted next-generation sequencing. In patients with germline *DDX41* variants, clinicopathologic features, co-occurring variants, variant allele frequency (VAF) distributions, and survival were analyzed. **Results:** *DDX41* variants were identified in 34/716 patients (4.7%), including 33 germline carriers (4.6%), occurring most frequently in AML (6.2%) and MDS (11.1%). Patients with germline *DDX41* variants had a median age of 68 years, were predominantly male (69.7%), and commonly had normal karyotypes (72.7%), with similar features in AML and MDS except for lower platelet counts in AML (*p* = 0.025). The most common germline *DDX41* variants were Y259C (20.6%), A500fs (14.7%), E7* (11.8%), V152G (11.8%), and D139G (8.8%), with null variants predominating in AML and missense variants in MDS (*p* = 0.002). Somatic *DDX41* variants occurred in 63.6% of patients with germline *DDX41* variants, with R525H being the most frequent (48%), while non-*DDX41* somatic variants were detected in 78.8% of patients, most commonly involving *ASXL1*, *DNMT3A*, *SRSF2*, and *TET2*, which appeared at lower VAFs, suggesting late acquisition. **Conclusions:** This study demonstrates ethnic-specific *DDX41* variant patterns in Korean myeloid neoplasm patients, with biallelic alterations potentially involved in early leukemogenesis. These findings provide further insight into the unique features of *DDX41*-associated myeloid neoplasms.

## 1. Introduction

Myeloid neoplasms with germline predisposition represent inherited genetic conditions that increase the risk of hematologic malignancies [1,2]. Identification of such predispositions is clinically important for accurate diagnosis, treatment decisions, donor selection for allogeneic hematopoietic stem cell transplantation, and genetic counseling for patients and their families.

DEAD-box RNA helicase 41 (*DDX41*) was first reported as a germline predisposition gene for myeloid neoplasms by Polprasert et al. in 2015 [3]. *DDX41*, located on chromosome 5q35.3, encodes a ubiquitously expressed 622-amino acid protein that plays essential roles in pre-mRNA splicing, RNA processing, innate immunity, and hematopoietic stem cell function [4]. Heterozygous germline variants in *DDX41* are now recognized as the most frequent germline predisposition to adult-onset myeloid neoplasms, particularly myelodysplastic neoplasm (MDS) and acute myeloid leukemia (AML) [5,6].

Clinically, *DDX41*-related myeloid neoplasms are characterized by late onset, male predominance, hypocellular marrow, and relatively favorable outcomes [7,8]. Ethnic differences in variant distribution have been reported, and the penetrance of germline *DDX41* variants has been estimated at around 62%, though it may vary by population and variant type [9]. Classifying germline *DDX41* variants remains challenging due to their heterogeneity and variable pathogenicity, often resulting in a substantial proportion being designated as variants of uncertain significance (VUS). To address this, several studies have proposed *DDX41*-specific modifications to the American College of Medical Genetics and Genomics/Association for Molecular Pathology (ACMG/AMP) guidelines [9,10,11,12]. Moreover, recent studies have demonstrated that patients with *DDX41* VUS exhibit clinical and molecular features comparable to those with pathogenic or likely pathogenic variants (PV/LPV), suggesting that many VUS may in fact be pathogenic and underscoring the need for refined classification frameworks [6,13].

Despite these advances, the biology and clinical impact of *DDX41*-mutated myeloid neoplasms are not yet fully understood. This study aims to investigate the frequency, spectrum, and clinical characteristics of *DDX41* variants in Korean patients with hematologic malignancies, including the patterns of co-occurring somatic variants.

## 2. Materials and Methods

### 2.1. Patients

This study included patients with hematologic malignancies–including AML, MDS, mixed phenotype acute leukemia (MPAL), chronic myeloid leukemia (CML), Philadelphia–negative myeloproliferative neoplasms (Ph-negative MPN), myelodysplastic/myeloproliferative neoplasms (MDS/MPN), and acute lymphoblastic leukemia (ALL)–who underwent targeted next-generation sequencing (NGS) between August 2020 and September 2024. All patients were diagnosed according to the 2016 World Health Organization (WHO) classification of hematolymphoid tumors [14] and were reclassified in this study based on the 2022 WHO classification or the International Consensus Classification (ICC) [1,2,15,16]. Laboratory data, including complete blood counts (CBC), bone marrow (BM) examinations, cytogenetic analyses (i.e., karyotyping and fluorescence in situ hybridization [FISH]), and molecular testing (i.e., targeted NGS and multiplex reverse transcription [RT]-PCR), along with clinical information such as treatment, disease course, and survival, were obtained from electronic medical records. For survival analysis, patients with AML aged ≥18 years who were diagnosed between July 2018 and December 2022 were used as control groups, including patients with AML with a normal karyotype (AML-NK) (*N* = 26), AML, myelodysplasia-related (AML-MR) (*N* = 58), and AML with mutated *TP53* (AML-*TP53*) (*N* = 33), with the latter two groups reclassified according to the ICC [2]. This study was approved by the Institutional Review Board of Samsung Medical Center (IRB No. 2025-03-023). The requirement for informed consent was waived due to the retrospective design of the study and the use of de-identified data.

### 2.2. Targeted NGS

Genomic DNA was extracted from BM aspirates or peripheral blood samples using the Promega DNA extraction kit (Promega, Madison, WI, USA) following the manufacturer’s instructions. Libraries were prepared using the IDT xGen predesigned/custom panel (Integrated DNA Technologies, Coralville, IA, USA) or the Twist custom somatic panel (Twist Bioscience, South San Francisco, CA, USA), targeting 49 hematologic malignancy-associated genes, including *DDX41* (Supplementary Table S1). Sequencing was performed on a NextSeq 550Dx instrument (Illumina, San Diego, CA, USA), as previously described [17]. Reads were aligned to the human reference genome GRCh37/hg19 using BWA-MEM (version 0.7.17). Mutect2 in the GATK package (v4.1.2) and Pisces (5.2.11.163) were used to detect single nucleotide variants (SNVs) and small insertion/deletions (indels), and Pindel (version 0.2.5b9) was used to detect large indels.

### 2.3. Classification of Germline DDX41 Variants

*DDX41* variants with variant allele frequency (VAF) ≥ 40% were considered to be germline origin, whereas those with VAF < 40% were presumed to be somatic in origin [11,12,18,19,20]. When available, germline origin was confirmed using cultured skin fibroblasts obtained from a skin punch biopsy. Germline *DDX41* variants were classified based on the ACMG/AMP guidelines [21,22], with a modified interpretation framework for *DDX41*, as proposed in recent studies [11,12], additionally applied. A pathogenic moderate criterion (PM1) was assigned to variants located within known functional domains of DDX41, including amino acid positions 181–209 (Q-motif), 212–396 (DEAD-box domain), 407–567 (helicase domain), and 580–597 (zinc finger [ZnF] domain) [12]. PM2 was applied to variants with minor allele frequency (MAF) < 0.008% in Genome Aggregation Database (gnomAD) or Korean Reference Genome Database (KRGDB) [11]. A pathogenic supporting criterion (PP3) was applied to variants with a rare exome variant ensemble learner (REVEL) score > 0.7 [11,12] or a SpliceAI delta score > 0.8 [23]. Importantly, PP4 with a very strong level (PP4_very strong) was applied when a germline *DDX41* variant was identified in conjunction with a presumed somatic *DDX41* variant [12]. Variants with a MAF ≥ 0.1% in gnomAD or KRGDB were classified as benign. In this study, *DDX41* variants classified as PV, LPV, and VUS were included for analysis.

### 2.4. Statistical Analysis

Categorical variables were compared using the chi-square or Fisher’s exact test, and continuous variables using the Mann–Whitney U, *t*-test, or Kruskal–Wallis test, as appropriate. The distribution of non-*DDX41* somatic VAFs was assessed with empirical cumulative distribution functions and compared using the Wilcoxon rank-sum test. Overall survival was analyzed from diagnosis to death or last follow-up using Kaplan–Meier estimates and log-rank tests, with pairwise comparisons adjusted by Bonferroni correction. Statistical analyses and visualization were performed using SPSS version (v29.0.2.0; IBM, Armonk, NY, USA) and R software (v4.5.1; R Foundation for Statistical Computing, Vienna, Austria). A *p*-value < 0.05 was considered statistically significant.

## 3. Results

### 3.1. Overall Frequency of DDX41 Variants

**Figure 1:**
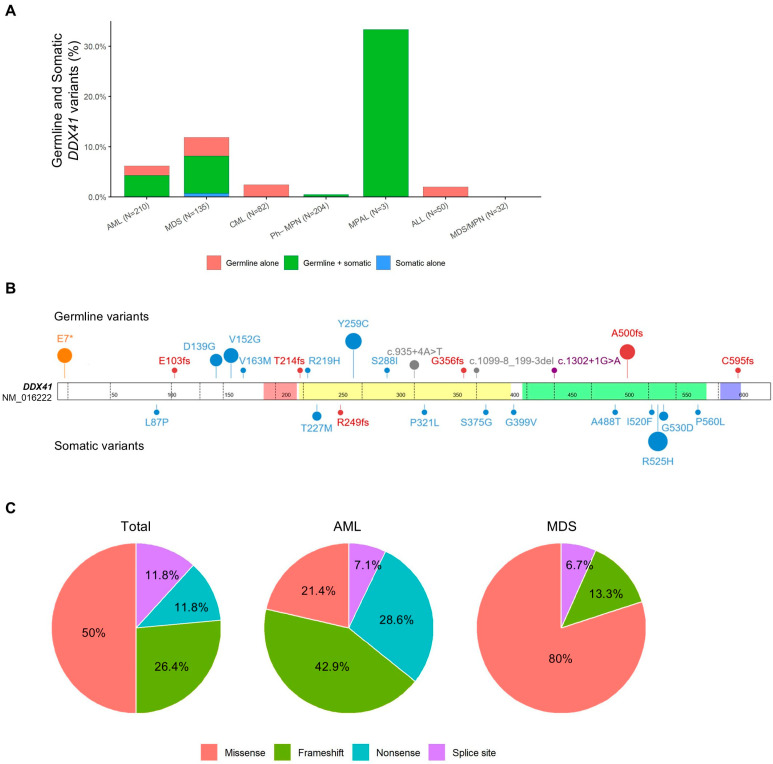
Frequency, distribution, and spectrum of *DDX41* variants in hematologic malignancies. (**A**) Frequency of germline and somatic *DDX41* variants by disease subtype. (**B**) Distribution of germline and somatic *DDX41* variants identified in this study. Variants are color-coded according to their type: missense (blue), frameshift (red), nonsense (orange), and splice site variants (purple and gray). The relative size of the circles reflects the number of patients carrying each variant. Functional domains are color-coded as follows: Q-motif (pink), DEAD-BOX domain (light yellow), helicase domain (light green), and ZnF domain (light purple). (**C**) Proportion of germline *DDX41* variant types in the total cohort, AML, and MDS patients.

*DDX41* variants were identified in 34 of 716 patients (4.7%) with hematologic malignancies, including 33 patients (4.6%) with germline *DDX41* variants and one with only a somatic *DDX41* variant (Supplementary Tables S2 and S3). The prevalence of germline *DDX41* variants varied by disease: 6.2% (13/210) in AML, 11.1% (15/135) in MDS, 2.4% (2/82) in CML, 0.5% (1/204) in Ph-negative MPN, 33.3% (1/3) in MPAL, and 2.0% (1/50) in ALL (Figure 1A). Co-occurring somatic *DDX41* variants were found in 63.6% (21/33) of patients with germline *DDX41* variants.

### 3.2. Clinical and Molecular Characteristics of Patients with Germline DDX41 Variants

Among 33 patients with germline *DDX41* variants, the median age at diagnosis was 68 years (range, 48–86 years), 69.7% (23/33) were male, and 72.7% (24/33) had a normal karyotype. Given that AML and MDS accounted for the majority of cases (28/33, 84.8%), comparative analyses were limited to these two subtypes to allow meaningful statistical comparison. Baseline characteristics (age, sex, hemoglobin, white blood cell counts, bone marrow cellularity, and normal karyotype frequency) showed no significant differences between AML and MDS patients with germline *DDX41* variants (all *p* > 0.05), except for lower platelet counts in AML (*p* = 0.025) (Table 1). The frequency of co-occurring somatic *DDX41* variant was also similar (*p* = 1.000). Among AML patients, 69.2% had AML-MR, while among MDS patients, 60% had MDS with low blasts, and 40% had MDS with increased blasts.

A total of 34 germline *DDX41* variants were identified, with eight confirmed as germline in cultured skin fibroblasts. One AML patient carried two germline variants (Glu7* and Cys595fs), both confirmed in fibroblasts. The median VAF of germline *DDX41* variants was 49.7% (interquartile range [IQR], 48.9–50.4%). When stratified by confirmation status, the median VAFs were 49.8% (range, 47.0–51.5%) for confirmed germline variants and 49.7% (range, 45.8–52.2%) for presumed germline variants, with no significant difference between groups (*p* = 0.496).

Of 15 unique germline variants, 73.3% (11/15) were classified as PV/LPV according to the modified ACMG/AMP criteria, while the remaining were classified as VUS (Supplementary Table S2). Among 33 patients with germline *DDX41* variants, six (18.2%) had VUS, all of which were missense variants without concomitant somatic *DDX41* variants. Clinical characteristics were broadly similar between patients with PV/LPV and those with VUS. Although patients with VUS showed higher WBC counts (median, 15.94 vs. 1.96 × 10^9^/L; *p* = 0.004) and higher BM cellularity (median, 80% vs. 20%; *p* = 0.003), other baseline features, including age, hemoglobin level, and karyotype status, did not differ significantly between groups (Supplementary Table S4). However, as each pathogenicity group comprised a heterogeneous mix of hematologic malignancy subtypes, baseline characteristics may be partly confounded by disease distribution.

The most prevalent germline variant was Y259C (20.6%), followed by A500fs (14.7%), E7* (11.8%), V152G (11.8%), and D139G (8.8%) (Figure 1B and Supplementary Table S5). Variant types differed significantly by disease type: null variants predominated in AML (78.6%), while missense variants were more common in MDS (80.0%) (*p* = 0.002) (Figure 1C).

### 3.3. Co-Occurring Somatic Variants and Their Allele Frequency Patterns

Somatic *DDX41* variants were identified in 22 patients (3.1%), with 25 variants comprising 11 unique ones. Two MDS patients had multiple somatic *DDX41* variants (two and three variants, respectively). All but one were missense variants, with a median VAF of 6.8% (IQR, 3.9–12.1%). The most frequent somatic variant was R525H (48.0%), followed by T227M, P321L, and G530D (8.0% each). Somatic *DDX41* variants were non-overlapping with germline *DDX41* variants (Figure 1B). While germline *DDX41* variants were distributed across the entire gene, somatic *DDX41* variants clustered in the DEAD-box and helicase C domains.

**Figure 2:**
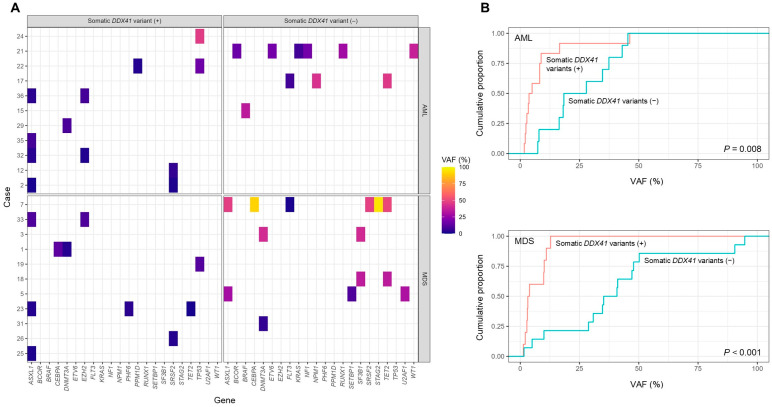
Spectrum and allele frequencies of non-*DDX41* somatic variants according to somatic *DDX41* variant status in AML and MDS patients with germline *DDX41* variants. (**A**) Heatmap showing the distribution and variant allele frequencies (VAFs) of non-*DDX41* somatic variants across individual cases, stratified by the presence or absence of somatic *DDX41* variants and by disease category (AML vs. MDS). Each tile represents a detected variant, with color intensity indicating the corresponding VAF (%). (**B**) Empirical cumulative distribution function (ECDF) plots of non-*DDX41* variant VAFs in AML (**top**) and MDS (**bottom**) patients, stratified by somatic *DDX41* variant status.

Non-*DDX41* somatic variants were detected in 78.8% (26/33) of patients with germline *DDX41* variants, including 84.6% of AML patients and 73.3% of MDS patients (*p* = 0.655). AML and MDS patients with somatic *DDX41* variants tended to have fewer non-*DDX41* somatic variants compared with those without (median 2, range 0–4 vs. median 3, range 0–8; *p* = 0.092). The most common co-occurring variants were *ASXL1* (32.1%), followed by *DNMT3A*, *SRSF2*, and *TET2* (14.3% each), and *EZH2* and *TP53* (10.7% each) (Figure 2A). To further evaluate VAF differences, we compared the distributions of non-*DDX41* variant VAFs between patients with and without somatic *DDX41* variants using empirical cumulative distribution function analysis. This demonstrated a significant leftward shift in both AML (*p* = 0.008) and MDS (*p* < 0.001) among patients without somatic *DDX41* variants, suggesting that co-occurring variants in the presence of somatic *DDX41* variants are more likely to occur at lower VAFs (Figure 2B).

### 3.4. Survival and Prognosis in AML and MDS with Germline DDX41 Variants

**Figure 3:**
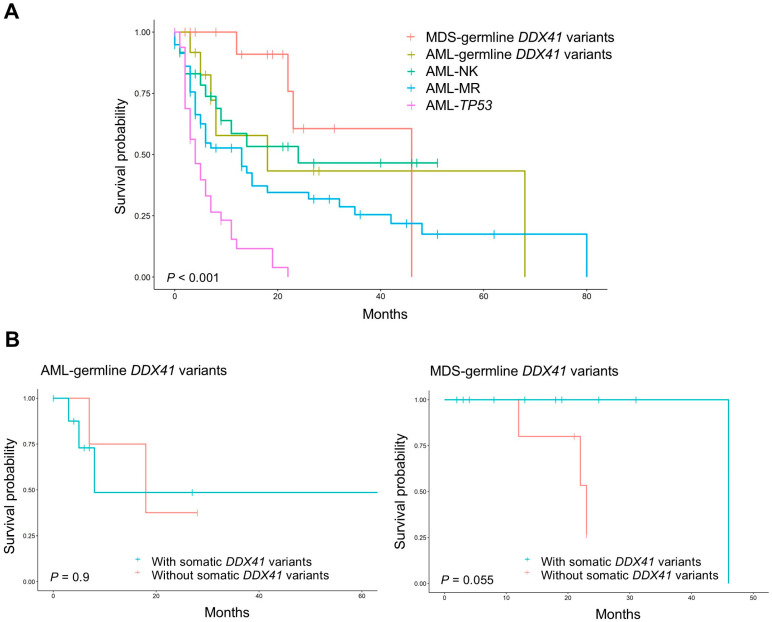
Survival analysis of patients with germline *DDX41* variants. (**A**) Overall survival of patients with MDS with germline *DDX41* variants (MDS-germline *DDX41*), AML with germline *DDX41* variants (AML-germline *DDX41*), AML with normal karyotype (AML-NK), AML with myelodysplasia-related changes (AML-MR), and AML with *TP53* mutations (AML-*TP53*). The overall survival differed significantly across groups (log-rank test, *p* < 0.001). Pairwise log-rank tests with Bonferroni correction showed significant differences between AML-*TP53* and all other groups (*p* < 0.05), while other pairwise comparisons did not reach statistical significance. (**B**) Survival of patients with AML-germline *DDX41* and MDS-germline *DDX41* according to the presence or absence of concomitant somatic *DDX41* variants.

The median follow-up period of AML and MDS patients with germline *DDX41* variants was 12.0 months (range, 0–68 months). Median survival was 18 months (95% CI, 7–not reached [NR]) for AML and 46 months (95% CI, 23–NR) for MDS. Survival was compared with AML subgroups: AML-NK, AML-MR, and AML-*TP53* (Figure 3A). AML-*TP53* had significantly worse survival than all other groups (*p* < 0.05), while differences among AML-NK, AML-MR, and AML/MDS with germline *DDX41* variants were not significant, likely due to small sample size. Notably, AML patients with germline *DDX41* variants, despite a high proportion of AML-MR (69.2%), showed survival patterns similar to AML-NK. In MDS, patients with concomitant somatic *DDX41* variants exhibited a trend toward better survival compared to those without (*p* = 0.055) (Figure 3B).

## 4. Discussion

This study investigated the frequency and spectrum of *DDX41* variants in Korean patients with hematologic malignancies, and evaluated the characteristics of somatic variants co-occurring with germline *DDX41* variants.

The incidence of *DDX41* variants in myeloid neoplasms has been reported to range from 2–5% across various studies [12,24], with a pooled incidence of 3.3% [25]. In the Korean population, a previous study identified germline *DDX41* variants in 6.1% of patients with AML, MDS, or idiopathic cytopenia of undetermined significance, with disease-specific frequencies of 2.3% in AML and 9.0% in MDS [26]. Another study reported an incidence of 2% in various hematologic malignancies, including AML, MDS, MPN, B-cell lymphoma, and multiple myeloma [11]. In our study, germline *DDX41* variants were detected in 4.6% of patients with hematologic malignancies, with higher prevalence in AML (6.2%) and MDS (11.1%). Among hematologic malignancies other than AML and MDS, *DDX41* variants have been rarely reported [11,20,25,27], and our study identified one patient with B-ALL, one with MPAL, two with CML, and one with PV.

Patients with germline *DDX41* variants in our study presented at a median age of 68 years, with male predominance (69.7%) and frequent normal karyotype (72.7%), demonstrating similar patterns in both AML and MDS. These characteristics represent hallmarks of *DDX41*-associated myeloid neoplasms [11,12,18,24,28]. The high normal karyotype rate suggests that disease progression may be driven by molecular rather than cytogenetic mechanisms. Moreover, the similarity in most baseline characteristics between AML and MDS patients with germline *DDX41* variants may point to a potential continuum model in which MDS could evolve into AML, with the lower platelet counts observed in AML possibly reflecting more advanced disease.

In this study, we included both PV/LPV and VUS *DDX41* variants. Similar to previous studies [6,13], patients with VUS exhibited clinical features that were largely comparable to those with PV/LPV variants, supporting the potential pathogenicity of these variants. However, because both groups encompassed heterogeneous hematologic malignancy subtypes, baseline differences may partly reflect disease distribution rather than inherent effects of variant classification. Further studies with larger and more homogeneous cohorts are needed to clarify these findings.

The spectrum of germline *DDX41* variants exhibits ethnic variability. In Western populations, common germline variants include M1? and D140fs [11,12], whereas Asian populations show higher frequencies of A500fs, Y259C, and V152G [26,28]. In Japanese patients, A500fs is most prevalent, followed by S363del, Y259C, and E7* [28]. In a prior Korean study, V152G was most common, followed by Y259C, A500fs, and E7* [26]. In our study, Y259C was most prevalent, followed by A500fs and V152G. Notably, the most common variants in Western populations, M1? and D140fs, were rarely observed in Japanese and Korean populations, whereas A500fs appeared to be Asian-specific and V152G was identified exclusively in Korean patients. These ethnic differences likely reflect founder effects and population-specific genetic architecture, underscoring the importance of ethnicity-informed genetic screening strategies. Furthermore, variant types showed disease-specific patterns, with null variants predominating in AML (78.6%) and missense variants in MDS (80%), suggesting that null variants may drive more aggressive disease phenotypes. This observation is consistent with the findings by Makishima et al., who reported that MDS patients with *DDX41* variants exhibited rapid progression to AML, with this aggressive transformation predominantly observed in those harboring null variants [28].

Concurrent somatic *DDX41* variants occur in approximately 50–87% of patients with germline *DDX41* variants in previous studies [3,11,12,18,26,28], and in 63.6% of patients with germline *DDX41* variants in our study. Maierhofer et al. reported a highly specific germline–somatic association (odds ratios > 350) and suggested that somatic *DDX41* variant presence can provide strong evidence for germline variant pathogenicity [12], thereby aiding VUS reclassification.

All somatic *DDX41* variants except one were missense variants, clustering within the DEAD-box and helicase domains, with R525H being the most frequent, followed by other recurrent variants including G530D, T227M, and P321L, consistent with previous reports [12,26,28]. Somatic *DDX41* variants alone are rare (0–0.6%) [11,18,26,28], and in our study, only one MDS patient (Case 9) harbored an isolated somatic *DDX41* frameshift variant (Arg249fs) caused by a 113-base pair insertion.

In our study, non-*DDX41* somatic variants were detected in 78.8% of patients with germline *DDX41* variants, with similar rates in AML and MDS. The most common variant was *ASXL1*, followed by *DNMT3A*, *SRSF2*, *TET2*, *EZH2*, and *TP53*, which often exhibited lower VAFs, suggesting late acquisition [12,18]. Co-occurrence of a *TP53* variant was relatively infrequent in our study, despite previous reports identifying it as the second most common co-occurring variant [24,25,29]. Patients with somatic *DDX41* variants had fewer additional somatic variants, and these non-*DDX41* variants occurred at significantly lower VAFs than in patients without somatic *DDX41* variants. Collectively, these findings suggest that biallelic *DDX41* alterations represent early leukemogenic events, thereby reducing the need for additional drivers [12,18], while non-*DDX41* variants tend to emerge later as subclonal events. Conversely, in the absence of biallelic *DDX41* alterations, leukemogenesis may require the acquisition of additional driver variants.

The prognostic impact of germline *DDX41* variants remains inconsistent across studies [3,11,20,24,26,30]. A recent study by Makishima et al. showed that standard prognostic scoring systems, such as the revised/molecular International Prognostic Scoring System (IPSS-R/M), were not predictive in *DDX41*-related myeloid malignancies [28]. Importantly, *DDX41* variants mitigated the poor prognosis typically associated with *TP53* co-variants, indicating a unique survival benefit. In our study, AML outcomes were similar to those of AML-NK, despite 69.2% being classified as AML-MR, while in MDS, concomitant somatic *DDX41* variants were associated with a trend toward better survival.

Our study has several limitations. Based on previous reports [11,12,18,19,20], we considered variants with a VAF ≥ 40% as germline in origin, and germline status was confirmed using cultured skin fibroblasts in only a subset of patients. Moreover, germline inference based solely on VAF carries inherent limitations, including the potential for confounding by copy number alterations or clonal hematopoiesis, although the latter is less likely at such high VAFs. We also included VUS, in addition to PV/LPV, to comprehensively evaluate their clinical relevance, although this may introduce some uncertainty in interpretation. In addition, our study lacked functional validation, such as protein expression or DNA damage marker analyses, which would help clarify the biological consequences of *DDX41* variants. Finally, the relatively small sample size and short follow-up period may limit the generalizability of our findings.

## 5. Conclusions

In conclusion, we characterized the frequency, spectrum, and clinical features of *DDX41* variants in Korean patients with hematologic malignancies. We found that concomitant somatic *DDX41* variants were associated with fewer co-occurring non-*DDX41* variants, which also tended to present at lower VAFs, supporting the role of biallelic *DDX41* alterations as early leukemogenic events. Our study contributes to a better understanding of the unique features of *DDX41*-associated myeloid neoplasms.

## Figures and Tables

**Table 1:** Clinical and laboratory characteristics of AML and MDS patients with germline *DDX41* variants.

Characteristics	AML (*N* = 13)	MDS (*N* = 15)	*p*
Median age, yrs (range)	68 (48–76)	69 (50–86)	0.406
Male, *N* (%)	9 (69.2)	11 (73.3)	1.000
Median Hb, g/dL (range)	8.3 (5.7–11.9)	8.8 (5.6–11.5)	0.747
Median WBC, ×10^9^/L (range)	1.72 (1.08–23.41)	2.21 (0.97–31.2)	0.213
Median PLT, ×10^9^/L (range)	47 (19–114)	101.5 (34–404)	0.025
Median BM blasts, % (range)	30.0 (19.8–92.3)	2.9 (0–18.0)	<0.001
Median BM cellularity, % (range)	20.0 (10–90)	22.5 (15–100)	0.950
Normal karyotype, *N* (%)	10 (76.9)	12 (80.0)	1.000
Any somatic mutation, *N* (%)	12 (92.3)	15 (100.0)	0.464
Somatic *DDX41* mutation	9 (69.2)	10 (66.7)	1.000
Other somatic mutation (excluding *DDX41*)	11 (84.6)	11 (73.3)	0.646
WHO classification, *N* (%)			
AML-MR	9 (69.2)		
AML with *KMT2A* rearrangement	1 (7.7)		
AML with *NPM1* mutation	1 (7.7)		
AML-NOS	2 (15.4)		
MDS-LB		7 (46.7)	
MDS-LB-*SF3B1*		1 (6.7)	
MDS-del(5q)		1 (6.7)	
MDS-IB1		3 (20.0)	
MDS-IB2		3 (20.0)	
Allogeneic HSCT, *N* (%)	3 (23.1)	3 (20.0)	1.000
Death, *N* (%)	6 (46.2)	4 (26.7)	0.433

Abbreviations: AML, acute myeloid leukemia; MDS, myelodysplastic neoplasm; yrs, years; Hb, hemoglobin; WBC, white blood cells; PLT, platelets; BM, bone marrow; WHO, world health organization; AML-MR, AML, myelodysplasia-related; AML-NOS, AML, not otherwise specified; MDS-LB, MDS with low blasts; MDS-LB-*SF3B1*, MDS with low blasts and *SF3B1* mutation; MDS-del(5q), MDS with low blasts and isolated 5q deletion; MDS-IB, MDS with increased blasts; HSCT, hematopoietic stem cell transplantation.

## Data Availability

All data relevant to the study have been included in the article or uploaded as Supplementary Information. The additional data presented in this study are available upon request from the corresponding author.

## Supplementary Materials

The following supporting information can be downloaded at: https://www.mdpi.com/article/10.3390/jcm14227999/s1, Table S1: List of genes included in the targeted next-generation sequencing panel; Table S2: Molecular and cytogenetic profiles of 34 patients with germline and/or somatic *DDX41* variants; Table S3: Frequency of germline and somatic *DDX41* variants in patients with hematologic malignancies; Table S4: Comparison of baseline characteristics of patients with germline *DDX41* variants by pathogenicity classification; Table S5: Frequency of germline *DDX41* variants by variant type.

## Abbreviations

The following abbreviations are used in this manuscript:

ALL	Acute lymphoblastic leukemia
AML	Acute myeloid leukemia
AML-MR	Acute myeloid leukemia, myelodysplasia-related
AML-NK	Acute myeloid leukemia with a normal karyotype
AML-*TP53*	Acute myeloid leukemia with *TP53* mutation
CBC	Complete blood counts
CML	chronic myeloid leukemia
DDX41	DEAD-box RNA helicase 41
FISH	Fluorescence in situ hybridization
LPV	Likely pathogenic variant
MAF	Minor allele frequency
MDS	Myelodysplastic neoplasm
MDS/MPN	Myelodysplastic/myeloproliferative neoplasm
MPAL	Mixed phenotype acute leukemia
MPN	Myeloproliferative neoplasm
NGS	Next-generation sequencing
PV	Pathogenic variant
SNV	Single nucleotide variant
VAF	Variant allele frequency
VUS	Variant of uncertain significance
