# Supplementary material for: Clinical and Molecular Spectrum of DDX41 Variants in Korean Patients with Hematologic Malignancies
Source: J Clin Med. 2025 Nov 11;14(22):7999. doi: 10.3390/jcm14227999 (PMC12653579; doi:10.3390/jcm14227999)
Supplement: Supplementary file 1 [file jcm-14-07999-s001.zip › jcm-3893505-supplementary.pdf]

**Supplementary Table S1.** List of genes included in the targeted next-generation sequencing panel.

| Gene | Reference sequence | Gene | Reference sequence | Gene | Reference sequence |
|---|---|---|---|---|---|
| *ABL1* | NM_005157.5 | *HRAS* | NM_001130442.2 | *SETBP1* | NM_015559.2 |
| *ANKRD26* | NM_014915.2 | *IDH1* | NM_005896.3 | *SF3B1* | NM_012433.3 |
| *ASXL1* | NM_015338.5 | *IDH2* | NM_002168.3 | *SH2B3* | NM_005475.2 |
| *BCOR* | NM_001123385.1 | *JAK2* | NM_004972.3 | *SRSF2* | NM_003016.4 |
| *BRAF* | NM_004333.4 | *KIT* | NM_000222.2 | *STAG2* | NM_001042749.2 |
| *CALR* | NM_004343.3 | *KRAS* | NM_033360.3 | *TERT* | NM_198253.2 |
| *CBL* | NM_005188.3 | *MPL* | NM_005373.2 | *TET2* | NM_001127208.2 |
| *CEBPA* | NM_004364.4 | *NF1* | NM_001042492.2 | *STAT3* | NM_139276.2 |
| *CSF3R* | NM_156039.3 | *NPM1* | NM_002520.6 | *PPM1D* | NM_003620.3 |
| *DDX41* | NM_016222.2 | *NRAS* | NM_002524.4 | *ELANE* | NM_001972.2 |
| *DKC1* | NM_001363.3 | *PHF6* | NM_032458.2 | *HAX1* | NM_006118.3 |
| *DNMT3A* | NM_022552.4 | *PIGA* | NM_002641.3 | *TP53* | NM_000546.5 |
| *ETV6* | NM_001987.4 | *PRPF8* | NM_006445.3 | *U2AF1* | NM_006758.2 |
| *EZH2* | NM_004456.4 | *PTPN11* | NM_002834.3 | *WT1* | NM_024426.4 |
| *FLT3* | NM_004119.2 | *RB1* | NM_000321.2 | *ZRSR2* | NM_005089.3 |
| *GATA1* | NM_002049.3 | *RUNX1* | NM_001754.4 | | |
| *GATA2* | NM_032638.4 | *SBDS* | NM_016038.2 | | |

**Supplementary Table S2.** Molecular and cytogenetic profiles of 34 patients with germline and/or somatic *DDX41* variants.

| Case No. | Sex | Age, yrs | Initial BM Diagnosis | Karyotype | Germline *DDX41* variants | | | | Somatic *DDX41* variants | | |
|---|---|---|---|---|---|---|---|---|---|---|---|
| | | | | | cDNA change | Protein change | VAF (%) | Variant classification[†] | cDNA change | Protein change | VAF (%) |
| 1 | M | 70 | MDS-LB | 46,XY[20] | [‡]c.1302+1G>A | p.? | 49.4 | LPV | c.962C>T | P321L | 12.1 |
| 2 | M | 68 | AML-MR | 46,XY[18] | c.308_309del | E103fs | 46.7 | PV | c.1574G>A | R525H | 5.7 |
| 3 | F | 78 | MDS-del(5q) | 47,XX,+8[11]/47,idem,del(5)(q22q35)[8]/46,XX[1] | c.776A>G | Y259C | 47.6 | PV | - | | |
| 4 | M | 67 | CML | 46,XY,t(9;22)(q34.1;q11.2)[20] | c.935+4A>T | p.? | 50.7 | PV | - | | |
| 5 | M | 64 | MDS-LB | 46,XY[20] | c.487G>A | V163M | 49.6 | VUS | - | | |
| 6 | M | 71 | MDS-IB1 | 46,XY[12] | c.776A>G | Y259C | 49.7 | PV | c.1574G>A, c.1679C>T | R525H, P560L | 10.7, 6.0 |
| 7 | M | 70 | MDS-IB2 | 45,X,–Y,+1,der(1;21)(q10;q10)[19]/46,XY[4] | c.656G>A | R219H | 50.8 | VUS | - | | |
| 8 | F | 67 | MDS-IB1 | 46,XX,ins(20;3)(q11.2;q21q26.2)[13]/46,XX[7] | - | | | | c.743_744ins113[§] | R249fs | 3.8 |
| 9 | F | 63 | PV-MF | 46,XX[13] | [‡]c.1099-8_1099-3del | p.? | 50.1 | PV | c.1574G>A | R525H | 1.4 |
| 10 | M | 68 | AML-MR | 46,XY[3] | c.19G>T | E7* | 50.1 | PV | c.1574G>A | R525H | 4.9 |
| 11 | M | 74 | MDS-LB | 46,XY[20] | c.776A>G | Y259C | 50.1 | PV | c.1574G>A | R525H | 8.9 |
| 12 | M | 49 | CML | 46,XY,t(9;22)(q34.1;q11.2)[20] | c.416A>G | D139G | 49.8 | VUS | - | | |
| 13 | M | 64 | AML with *KMT2A* rearrangement | 50,XY,+4,+8,der(10)t(10;11)(p12;q23)inv(11)(q23q13),der(11)t(10;11)(p12;q13),+16,+20[20] | c.416A>G | D139G | 46.1 | VUS | - | | |
| 14 | F | 48 | B-ALL, NOS | 46,XX,del(11)(q13q23)[10]/46,idem,del(13)(q12q22)[8]/46,XX[3] | c.455T>G | V152G | 49.6 | PV | - | | |
| 15 | M | 66 | AML with *NPM1* mutation | 46,XY[20] | c.863_864delinsTT | S288I | 49.2 | VUS | - | | |
| 16 | F | 86 | MDS-LB-*SF3B1* | 46,XX[20] | c.416A>G | D139G | 50.4 | VUS | - | | |
| 17 | M | 60 | MDS-IB2 | 45,XY,–2,–5,–7,–18,+3mar,inc[4]/46,XY[16] | c.776A>G | Y259C | 49.9 | PV | c.680C>T | T227M | 6.5 |

| | | | | | | | | | | | |
|---|---|---|---|---|---|---|---|---|---|---|---|
| 18 | F | 69 | MDS-LB | 46,XX[4] | c.776A>G | Y259C | 52.2 | PV | c.1123A>G | S375G | 19.6 |
| 19 | F | 48 | AML-MR | 46,XX[2] | c.1496dup | A500fs | 50.8 | PV | - | | |
| 20 | M | 69 | AML-MR | 45,XY,del(5)(q22q31),–8,der(17)t(2;17)(p12;p13),der(20)t(8;20)(q21.3;q11.2)[8]/46,XY[12] | c.776A>G | Y259C | 50.2 | PV | c.1574G>A | R525H | 19.4 |
| 21 | F | 75 | MDS-LB | 46,XX[1] | c.455T>G | V152G | 49.5 | PV | c.962C>T, c.1196G>T, c.1574G>A | P321L, G399V, R525H | 1.3, 3.9, 1.8 |
| 22 | M | 48 | AML-MR | 46,XY,add(17)(p11.2)[10] | c.19G>T | E7* | 45.8 | PV | c.1558A>T | I520F | 36.3 |
| 23 | M | 62 | MDS-LB | 46,XY[20] | c.776A>G | Y259C | 49.3 | PV | c.1589G>A | G530D | 31.8 |
| 24 | M | 62 | MDS-IB2 | 46,XY[20] | ‡c.1067del | G356fs | 47.0 | PV | c.1574G>A | R525H | 3.8 |
| 25 | F | 74 | AML, NOS | 46,XX[17] | c.935+4A>T | p.? | 50.4 | PV | c.1574G>A | R525H | 6.8 |
| 26 | M | 72 | MPAL, M/B | 46,XY[20] | ‡c.1496dup | A500fs | 50.8 | PV | c.1462G>A | A488T | 10.3 |
| 27 | M | 64 | AML, NOS | 46,XY[6] | ‡c.19G>T, ‡c.1784del | E7*, C595fs | 49.1, 48.9 | PV, LPV | c.260T>C | L87P | 7.9 |
| 28 | M | 50 | MDS-LB | 46,XY[20] | c.455T>G | V152G | 49.0 | PV | c.680C>T | T227M | 13.8 |
| 29 | M | 82 | MDS-IB1 | 46,XY[20] | c.1496dup | A500fs | 47.4 | PV | - | | |
| 30 | M | 76 | AML-MR | 46,XY[20] | c.639del | T214fs | 48.0 | PV | c.1574G>A | R525H | 4.2 |
| 31 | M | 64 | MDS-IB1 | 46,XY[20] | c.455T>G | V152G | 50.5 | PV | c.1574G>A | R525H | 12.4 |
| 32 | F | 65 | AML-MR | 46,XX[4] | ‡c.1496dup | A500fs | 51.5 | PV | - | | |
| 33 | F | 69 | AML-MR | 46,XX[20] | ‡c.1496dup | A500fs | 50.7 | PV | c.1589G>A | G530D | 7.8 |
| 34 | M | 73 | AML-MR | 46,XY[20] | c.19G>T | E7* | 47.5 | PV | c.1574G>A | R525H | 3.5 |

†A modified ACMG/AMP guideline was adopted.

‡Germline origin was confirmed using cultured skin fibroblasts.

§c.743_744insAGCTCTCCCAACAGTCTATCTGGCCGTGACATGATAGGCATCGCTTTCACGGGTTCAGGCAAGACACTGGTGTTCACGTTGCCCGTCATCATGTTCTGCCTGGAACAAGAGAA GGAACAAGAGAA

Abbreviations: F, female; M, male; yrs, years; AML, acute myeloid leukemia; AML-MR, AML with myelodysplasia related; NOS, not otherwise specified; CML, chronic myeloid leukemia; MDS, myelodysplastic neoplasm; MDS-LB, MDS with low blasts; MDS-IB, MDS with increased blasts; MDS-del(5q), MDS with low blasts and isolated 5q deletion; MDS-LB-SF3B1, MDS with low blasts and *SF3B1* mutation; ALL, acute lymphoblastic leukemia; PV, polycythemia vera; PV-MF, post-PV-myelofibrosis; MPAL, mixed phenotype acute leukemia; VAF, variant allelic frequency; PV, pathogenic variant; LPV, likely pathogenic variant; VUS, variant of uncertain significance.

**Supplementary Table S3.** Frequency of germline and somatic *DDX41* variants in patients with hematologic malignancies.

| Germline vs. Somatic *DDX41* Variants | AML | MDS | CML | $Ph^{-}$ MPN | MPAL | ALL | MDS/MPN | Total |
|---|---|---|---|---|---|---|---|---|
| Patients, N | 210 | 135 | 82 | 204 | 3 | 50 | 32 | 716 |
| Any *DDX41* variants, N (%) | 13 (6.2) | 16 (11.9) | 2 (2.4) | 1 (0.5) | 1 (33.3) | 1 (2.0) | 0 (0.0) | 34 (4.7) |
| Germline *DDX41* variants, N (%) | 13 (6.2) | 15 (11.1) | 2 (2.4) | 1 (0.5) | 1 (33.3) | 1 (2.0) | 0 (0.0) | 33 (4.6) |
| Germline + Somatic *DDX41* variants, N (% of germline*) | 9 (69.2) | 10 (66.7) | 0 (0.0) | 1 (100.0) | 1 (100.0) | 0 (0.0) | 0 (0.0) | 21 (63.6) |
| Somatic *DDX41* variants, N (%) | 9 (4.3) | 11 (8.1) | 0 (0.0) | 1 (0.5) | 1 (33.3) | 0 (0.0) | 0 (0.0) | 22 (3.1) |

*Percentage among patients with germline *DDX41* variants

Abbreviations: AML, acute myeloid leukemia; MDS, myelodysplastic neoplasm; CML, chronic myeloid leukemia; $Ph^{-}$ MPN, Philadelphia–negative myeloproliferative neoplasm; MPAL, mixed phenotype acute leukemia; ALL, acute lymphoblastic leukemia; MDS/MPN, myelodysplastic/myeloproliferative neoplasm.

**Supplementary Table S4.** Comparison of baseline characteristics of patients with germline *DDX41* variants by pathogenicity classification.

| Characteristics | Germline *DDX41* variants | | *P* |
|---|---|---|---|
| | PV/LPV (N=27) | VUS (N=6) | |
| Median age, yrs (range) | 68 (48–76) | 66 (64–86) | 0.833 |
| Male, N (%) | 18 (66.7) | 5 (83.3) | 0.640 |
| Median Hb, g/dL (range) | 8.5 (5.6–12.9) | 8.1 (7.7–9.9) | 0.825 |
| Median WBC, $\times 10^9$/L (range) | 1.96 (1.08–29.62) | 15.94 (2.35–31.2) | 0.004 |
| Median PLT, $\times 10^9$/L (range) | 78 (19–719) | 44 (38–360) | 0.779 |
| Median BM blasts, % (range) | 17.4 (0–91) | 10 (0–92) | 0.455 |
| Median BM cellularity, % (range) | 20 (10–100) | 80 (70–100) | 0.003 |
| Normal karyotype, N (%) | 21 (77.8) | 3 (50.0) | 0.309 |
| Any somatic mutation, N (%) | 25 (92.6) | 6 (100) | 1.000 |
| Somatic *DDX41* mutation | 21 (77.8) | 0 | 0.001 |
| Other somatic mutation (excluding *DDX41*) | 20 (74.1) | 6 (100) | 0.301 |
| Hematologic malignancy subtype, N (%) | | | 0.719 |
| AML | 11 (40.7) | 2 (33.3) | |
| MDS | 12 (44.4) | 3 (50.0) | |
| CML | 1 (3.7) | 1 (16.7) | |
| PV | 1 (3.7) | 0 | |
| MPAL | 1 (3.7) | 0 | |
| ALL | 1 (3.7) | 0 | |
| Allogeneic HSCT, N (%) | 7 (25.9) | 1 (16.7) | 1.000 |
| Death, N (%) | 6 (22.2) | 4 (66.7) | 0.053 |

Abbreviations: PV, pathogenic variant; LPV, likely pathogenic variant; VUS, variant of uncertain significance; yrs, years; Hb, hemoglobin; WBC, white blood cells; PLT, platelets; BM, bone marrow; AML, acute myeloid leukemia; MDS, myelodysplastic neoplasm; CML, chronic

myeloid leukemia; PV, polycythaemia vera; MPAL, mixed phenotype acute leukemia; ALL, acute lymphoblastic leukemia; HSCT, hematopoietic stem cell transplantation.

**Supplementary Table S5.** Frequency of germline *DDX41* variants by variant type.

| Germline variants | AML | MDS | CML | Ph⁻ MPN | MPAL | ALL | Total |
|---|---|---|---|---|---|---|---|
| Missense variants, N (%) | 3 (21.4) | 12 (80.0) | 1 (50.0) | 0 (0.0) | 0 (0.0) | 1 (100.0) | 17 (50.0) |
| c.776A>G, p.Y259C | 1 | 6 | 0 | 0 | 0 | 0 | 7 |
| c.455T>G, p.V152G | 0 | 3 | 0 | 0 | 0 | 1 | 4 |
| c.416A>G, p.D139G | 1 | 1 | 1 | 0 | 0 | 0 | 3 |
| c.487G>A, p.V163M | 0 | 1 | 0 | 0 | 0 | 0 | 1 |
| c.656G>A, p.R219H | 0 | 1 | 0 | 0 | 0 | 0 | 1 |
| c.863_864delinsTT, p.S288I | 1 | 0 | 0 | 0 | 0 | 0 | 1 |
| Null variants, N (%) | 11 (78.6) | 3 (20.0) | 1 (50.0) | 1 (100.0) | 1 (100.0) | 0 (0.0) | 17 (50.0) |
| c.1496dup, p.A500fs | 3 | 1 | 0 | 0 | 1 | 0 | 5 |
| c.19G>T, p.E7* | 4 | 0 | 0 | 0 | 0 | 0 | 4 |
| c.935+4A>T, p.? | 1 | 0 | 1 | 0 | 0 | 0 | 2 |
| c.308_309del, p.E103fs | 1 | 0 | 0 | 0 | 0 | 0 | 1 |
| c.639del, p.T214fs | 1 | 0 | 0 | 0 | 0 | 0 | 1 |
| c.1067del, p.G356fs | 0 | 1 | 0 | 0 | 0 | 0 | 1 |
| c.1099-8_1099-3del, p.? | 0 | 0 | 0 | 1 | 0 | 0 | 1 |
| c.1302+1G>A, p.? | 0 | 1 | 0 | 0 | 0 | 0 | 1 |
| c.1784del, p.C595fs | 1 | 0 | 0 | 0 | 0 | 0 | 1 |

Abbreviations: AML, acute myeloid leukemia; MDS, myelodysplastic neoplasm; CML, chronic myeloid leukemia; Ph⁻ MPN, Philadelphia–negative myeloproliferative neoplasm; MPAL, mixed phenotype acute leukemia; ALL, acute lymphoblastic leukemia; MDS/MPN, myelodysplastic/myeloproliferative neoplasm.
